# Investigating Seed Germination, Seedling Growth, and Enzymatic Activity in Onion (*Allium cepa*) Under the Influence of Plasma-Treated Water

**DOI:** 10.3390/ijms26157256

**Published:** 2025-07-27

**Authors:** Sabnaj Khanam, Young June Hong, Eun Ha Choi, Ihn Han

**Affiliations:** 1 Department of Plasma Bio-Display, Kwangwoon University, Seoul 01897, Republic of Korea; sabnajkhanam719@gmail.com; 2 Department of Electrical and Biological Physics, Kwangwoon University, Seoul 01897, Republic of Korea; 3 Advanced Technology Research Institute, Nayuda Co., Seoul 04067, Republic of Korea; 4 Dasanje 301, Kwangwoon ro 20, Nowongu, Seoul 01890, Republic of Korea

**Keywords:** seed germination, cylindrical dielectric barrier discharge (c-DBD) plasma, reactive nitrogen species (RNS), plasma-treated water (PTW), onion (*Allium cepa*)

## Abstract

Seed germination and early seedling growth are pivotal stages that define crop establishment and yield potential. Conventional agrochemicals used to improve these processes often raise environmental concerns, highlighting the need for sustainable alternatives. In this study, we demonstrated that water treated with cylindrical dielectric barrier discharge (c-DBD) plasma, enriched with nitric oxide (NO) and reactive nitrogen species (RNS), markedly enhanced onion (*Allium cepa*) seed germination and seedling vigor. The plasma-treated water (PTW) promoted rapid imbibition, broke dormancy, and accelerated germination rates beyond 98%. Seedlings irrigated with PTW exhibited significantly increased biomass, root and shoot length, chlorophyll content, and antioxidant enzyme activities, accompanied by reduced lipid peroxidation. Transcriptomic profiling revealed that PTW orchestrated a multifaceted regulatory network by upregulating gibberellin biosynthesis genes (*GA3OX1/2*), suppressing abscisic acid signaling components (*ABI5*), and activating phenylpropanoid metabolic pathways (*PAL, 4CL*) and antioxidant defense genes (*RBOH1*, *SOD*). These molecular changes coincided with elevated NO_2_^−^ and NO_3_^−^ levels and finely tuned hydrogen peroxide dynamics, underpinning redox signaling crucial for seed activation and stress resilience. Our findings establish plasma-generated NO-enriched water as an innovative, eco-friendly technology that leverages redox and hormone crosstalk to stimulate germination and early growth, offering promising applications in sustainable agriculture.

## 1. Introduction

Onion (*Allium cepa* L.) is one of the most globally important vegetable crops, ranking second only to tomato in terms of production volume [1]. According to the Food and Agriculture Organization (FAO), global onion production reached 110 million tons across more than 5.9 million hectares of agricultural land in 2024 (FAO, 2024). Within the European Union (EU), approximately 6.8 million tons of dry onions were harvested from 178,000 hectares, with Spain remaining the top producer, yielding 1.35 million tons from 25,500 hectares (FAOSTAT, 2024). This widespread cultivation reflects the crop’s versatility and strong domestic and international demand. In addition to its large cultivation area and high yield, *A. cepa* holds considerable global and regional economic significance. The global onion market was valued at approximately USD 50.5 billion in 2023 and is projected to surpass USD 60 billion by 2030, driven by rising consumption in households, food processing industries, and traditional medicine sectors [1]. India (28.6%) and China (22.2%) are the leading producers, together accounting for over 50% of the world’s total onion production, followed by Egypt (3.3%), the United States (2.64%), Bangladesh (2.3%), and Turkey (2.1%), while other countries contribute less than 1% individually [2,3]. Regionally, the European Union represents a significant market, with onions contributing substantially to the vegetable sector’s economic output. For example, the EU onion trade (exports and imports combined) accounted for over EUR 1 billion annually in recent years [1], supporting both large-scale agribusiness and smallholder farmers. These figures underscore *A. cepa*’s role not only as a dietary staple but also as a key agricultural commodity with increasing market relevance.

Furthermore, onions play a crucial role in diverse cuisines worldwide and are consumed in various forms, including fresh, cooked, and dried, contributing to a wide range of dishes such as soups, stews, sauces, and salads [2,3]. In Republic of Korea, onions are commonly used in broths and side dishes. Their popularity over the past five decades can be attributed to their unique flavor, culinary flexibility, long shelf life, and nutritional value [4]. Nutritionally, onions are rich in essential components like fiber, carbohydrates, vitamin C, potassium, and folate [5]. They also contain bioactive compounds such as quercetin, *p*-coumaric acid, flavonoids, and sulfur-containing compounds like allicin, which are associated with antioxidant, anti-inflammatory, and antimicrobial activities [2,6]. These properties make onions not only a dietary staple but also a functional food with established health benefits.

To meet the increasing demand for onions, many farmers utilized intensive agricultural practices, including the excessive use of synthetic fertilizers and chemical growth enhancers [7,8]. Although these methods can temporarily boost productivity, they often result in serious environmental consequences, including soil degradation, nitrate pollution of water bodies, disruption of beneficial soil microbiota, and increased greenhouse gas emissions [9]. These challenges highlight the urgent need for sustainable agricultural technologies that maintain crop productivity while minimizing ecological damage.

In response to these concerns, plasma-based technologies have emerged as innovative and eco-friendly alternatives to conventional agrochemicals [10]. Plasma-treated water (PTW) is one such approach, created by exposing water to nonthermal biocompatible plasma, which generates reactive oxygen and nitrogen species (RONS) such as hydrogen peroxide (H_2_O_2_), nitrate (NO_3_^−^), nitrite (NO_2_^−^), and hydroxyl radicals (•OH) [11,12,13,14]. These reactive species have been shown to positively influence various physiological processes in plants, including improved seed germination, enzymatic activities, nutrient uptake, and stress tolerance [15]. Previous studies have demonstrated the beneficial effects of PTW on crops like lettuce, wheat, rice, maize, tomato, and spinach, promoting seed germination, root and shoot development, biomass accumulation, and the activation of antioxidant enzymes [14,15,16,17]. These effects are often attributed to enhanced cellular signaling, defense responses, and metabolic regulation [13]. However, the impact of PTW on *Allium cepa*, particularly during the early stages of growth, remains underexplored.

Therefore, this study aims to investigate the potential of plasma-treated water as a sustainable method to enhance onion growth. Specifically, it examines PTW’s effects on seed germination, seedling development (root and shoot length), the activity of antioxidant enzymes such as catalase and peroxidase, and the expression of genes associated with protein synthesis. The findings are expected to contribute to the advancement of sustainable agricultural practices by reducing reliance on chemical inputs while supporting high crop yields.

## 2. Results

### 2.1. Characterization of Multi-Electrode Cylindrical Dielectric Barrier Discharge (c-DBD) Plasma System

The discharge voltage and current were measured using an oscilloscope equipped with a high-voltage probe (1:1000) and a current probe, with the plasma device setup as shown in 2.13 section The discharge power and its corresponding formula include the integral of the time-varying voltage and current over the applied frequency, as shown in Equation (1).

(1) $$P=freqency\times \int_{0}^{T}V(t)\cdot I\left(t\right)dt$$

**Figure 1:**
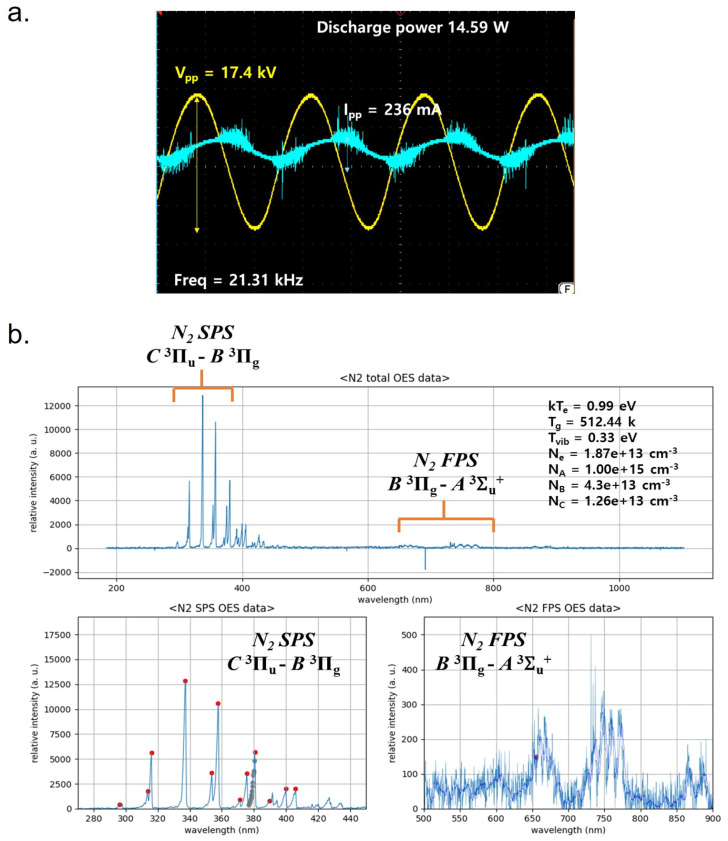
(**a**) Discharge voltage and current of multi-electrode c-DBD plasma; (**b**) plasma temperature and density of c-DBD plasma.

The c-DBD plasma used continuous pulses, unlike burst pulses, to generate high concentrations of nitric oxide gas in the plasma. The peak-to-peak voltage and current was about 17.40 kV and 236 mA, respectively. The frequency was 21.31 kHz. In these devices, the spike-shaped signals did not appear, fine current peak waveforms were observed during the period of plasma discharge, as shown in Figure 1a. Figure 1b displays the optical emission spectra, which revealed the presence of several excited nitrogen species. These included the molecular nitrogen first positive system (FPS, B^3^Π_g → A^3^Σ_u), first negative system (FNS, B^2^Σ_g^+^ → X^2^Σ_g^+^), and second positive system (SPS, C^2^Π_u → B^3^Π_g). The emission wavelength ranging from 650 to 800 nm was attributed to the N_2_ FPS, whereas 300–400 nm represents SPS emission bands [15,16,17]. The gas-FTIR result confirmed different types of reactive nitrogen species were formed, such as NO, NO_2_, and N_2_O. Figure S1a,b showed NO concentration exhibits a sharp rise and reaches a plateau after approximately 10 min. N_2_O concentration initially increases but then decreases and stabilizes, forming a stable curve. NO_2_ concentration gradually increases but remains below 40% of the NO concentration throughout the entire measurement period. NO concentration was approximately 1000 ppm. The NO_2_ concentration was 400 ppm. Additionally, four pairs of electrodes were used in this experiment, and plasma generation area length is 100 mm.

### 2.2. Water Uptake

**Figure 2:**
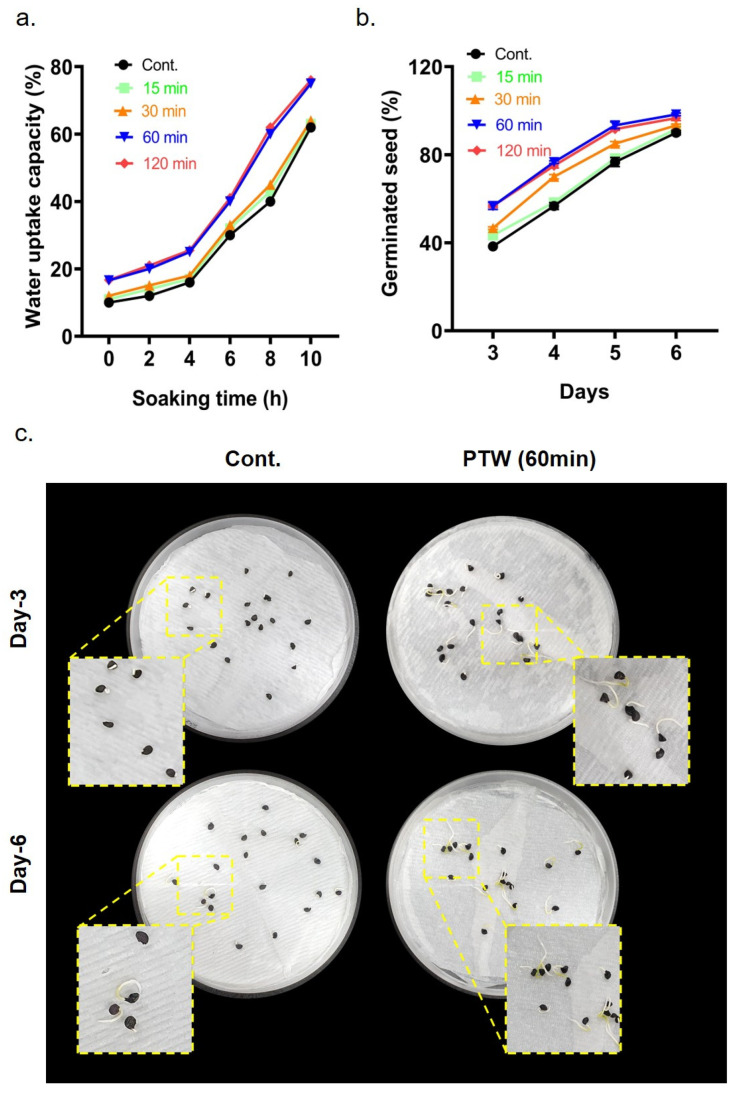
Effects of c-DBD plasma treatment on the seed germination of onion. (**a**) Water uptake capacity in onion seeds after different soaking durations. (**b**) Seed germination monitored over 3–6 days following 15–120 min of plasma treatment. (**c**) Photographs showing germination status of seeds (days 3 and 6) following 60 min of plasma treatment.

This study examined the water absorption characteristics of seeds exposed to deionized water (DW) and plasma-treated water for different durations over a period of 10 h. The water absorption is essential for initiating seed germination [18]. As depicted in Figure 2a, onion seeds soaked in PTW, prepared for various treatment times at intervals of 0, 2, 4, 6, 8, and 10 h, showed a continued increase up to 8 h with no significant rise thereafter; 60 and 120 min showed the highest percentage of water uptake in Figure 2a.

### 2.3. Determination of Seed Germination

This study explored the influence of dielectric barrier discharge c-DBD treatment on the germination of onion seeds (Figure 2b). Sterilized dry seeds of onion were exposed to plasma for 15, 30, 60, and 120 min, then placed in a growth chamber to germinate. The findings revealed that plasma-treated seeds exhibited significantly higher germination compared to untreated seeds, particularly noticeable within 3 to 6 days after treatment (Figure 2b,c). In addition, 60 min of c-DBD plasma exposure resulted in a higher seed germination rate, around 98.33%, compared to the control and other treated groups, as shown in Figure 2b,c. Based on these results, control water and60 min plasma-treated water were selected for all subsequent experiments.

### 2.4. Growth Related Variables

**Figure 3:**
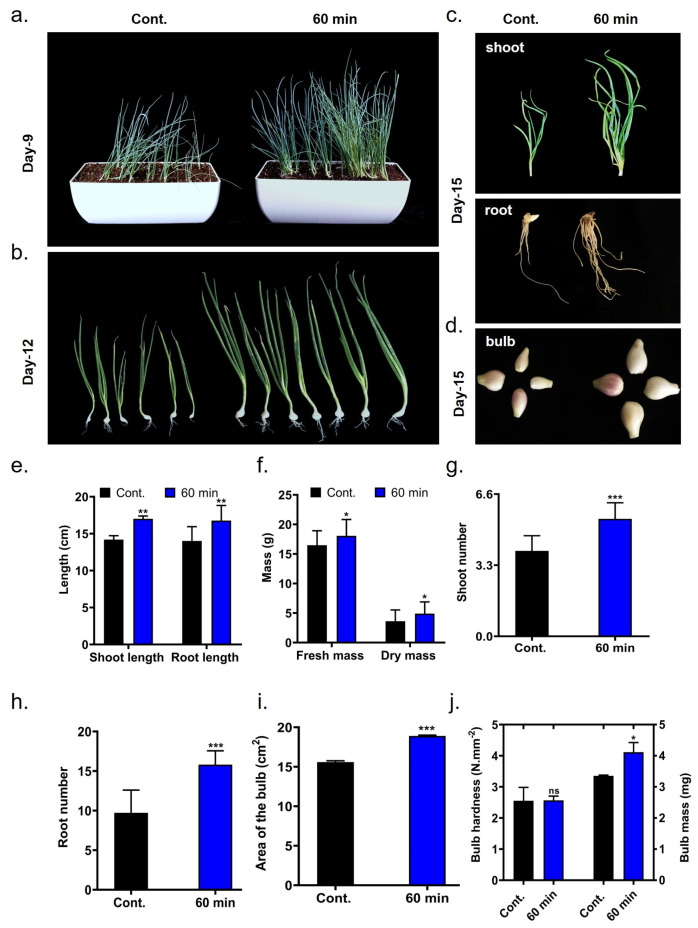
Effect of c-DBD plasma treatment on onion seedlings growth. (**a**) Shoot morphology at 9days, (**b**) whole seedling length at 12 days, (**c**) shoot and root length at 15 days, (**d**) bulb morphology, (**e**) total length (shoot and root), (**f**) weight (fresh and dry mass), (**g**) shoot number, (**h**) root number, (**i**) area of the bulb, (**j**) bulb hardness and mass. Statistical differences between treatment groups are indicated as * *p* < 0.05, ** *p* < 0.01, and *** *p* < 0.001 and “ns” denoting no significant difference compared to the control group.

Seedling growth-related variables, including overall length, weight (fresh and dry mass), and shoot and root number of onion seedlings, were examined for both control and the 60 min plasma-treated group after 9–15 days of cultivation, as presented in Figure 3. As shown in Figure 3a–c,e,f, 60 min of plasma treatment led to a substantial increase in length and weight compared to the untreated control. Shoot and root length values were 17.01 and 16.77 cm, respectively, in the plasma-treated group, compared to 14.18 and 14.01 cm in the control group. Similarly, fresh and dry mass were increased in the plasma-treated group (18.06 and 4.91 g) compared to the control (14.68 and 3.41), as shown in Figure 3f. Notably, shoot and root numbers also improved in the plasma-treated group (Figure 3g,h). Figure 3i shows that the total area of the onion bulb was not adversely affected by plasma treatment(18.90 cm^2^) in the plasma-treated group vs. in the control (15.59 cm^2^). The plasma-treated group exhibited increased bulb hardness and mass relative to the control (Figure 3j).

### 2.5. Chlorophyll Content

**Figure 4:**
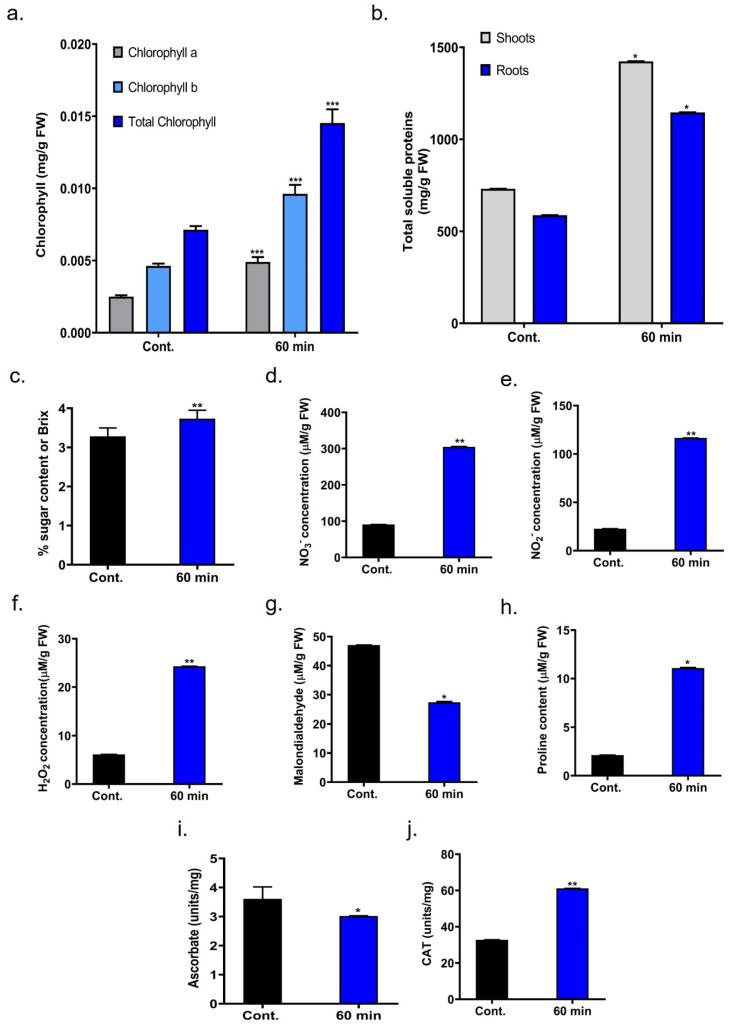
The biochemical analysis of onion seedlings under control and 60 min of c-DBD plasma treatment. (**a**) Chlorophyll, (**b**) total soluble proteins, (**c**) sugar content, (**d**) NO_3_^−^, (**e**) NO_2_^−^, (**f**) H_2_O_2_ concentration, (**g**) malondialdehyde, (**h**) proline, (**i**) ascorbate, and (**j**) catalase activity. Statistical differences between treatment groups are indicated as * *p* < 0.05, ** *p* < 0.01, and *** *p* < 0.001 and “ns” denoting no significant difference compared to the control group.

Chlorophyll a, chlorophyll b, and total chlorophyll are key pigments that play a crucial role in absorbing light for photosynthesis. When compared to the control, chlorophyll a level increased by 1.96-fold with 60 min of plasma treatment. A similar trend was observed for chlorophyll b, which rose by 2.08-fold in the respective treatment group by day 12. Total chlorophyll content also improved significantly, showing a 2.04-fold increase in the plasma-treated group (Figure 4a).

### 2.6. Total Soluble Protein and Sugar Content in Onion

Effects of c-DBD plasma treatment on the levels of total soluble proteins in the onion shoots and roots. Total soluble protein (TSP) levels generally showed an upward trend in the plasma-treated group than in the untreated control. The highest TSP concentration in shoot was approximately 1423.75 mg/g, whereas root was 1146.25 mg/g, as shown in Figure 4b. The sugar content of the onion seedling was approximately 3.73% in the plasma-treated group, compared to a 3.28% reduction in the untreated control group (Figure 4c).

### 2.7. c-DBD Plasma Induced Reactive Species

Reactive nitrogen species (RNS) accumulation in onion seedlings was measured using in situ and colorimetric techniques. NO_2_^−^ levels were significantly increased by c-DBD plasma treatment (116.57 µM/g FW) compared to the control (22.75 µM/g FW), as shown in Figure 4d. Similarly, in Figure 4e, NO_3_^−^ levels on day 12 exhibited a notable increase of 304.97 µM/g FW in the 60 min plasma-treated group, compared to 90.93 µM/g FW in the control. The highest concentration of H_2_O_2_ (24.29 µM/g FW) was detected in onion seedlings from the 60 min plasma-treated group (Figure 4f).

### 2.8. Malondialdehyde (MDA Content

Malondialdehyde (MDA), a well-recognized marker of oxidative damage, was measured to assess membrane disruption caused by reactive oxygen species generated from c-DBD plasma treatment. The findings revealed a significant decline in MDA levels in onion seedlings treated with c-DBD plasma-treated water. MDA levels were reduced to 58% in the 60 min plasma-treated group compared to the control, as shown in Figure 4g. These results indicate that c-DBD-treated water not only alleviates oxidative damage but also promotes the accumulation of beneficial biomolecules such as chlorophyll and soluble protein.

### 2.9. Determination of Proline Content

Under different types of environmental stress, plants often accumulate proline, an amino acid that acts as an osmo-protectant, scavenges hydroxyl radicals, regulates osmotic pressure, and protects enzyme function. In this study, proline content rose substantially by 5.22-fold compared to the control (Figure 4h).

### 2.10. Asessment of Ascorbate Content

The small antioxidant molecule ascorbate functions as a critical detoxifying substance which protects cells from hydrogen peroxide (H_2_O_2_). This finding confirmed that treatment with plasma-treated water reduced the ascorbate content to 83% in 60 min periods of seedlings compared to untreated controls (Figure 4i).

### 2.11. Evalution of Catalase Activity

The enzyme catalase (CAT) defends cells by decomposing H_2_O_2_ into water together with oxygen. Recovery of the antioxidative system was observed after 60 min of plasma application, as evidenced by increased CAT enzyme activity. CAT activity increased 1.86-fold in the treated group compared to the control, as shown in Figure 4j.

### 2.12. Gene Expression Alters by c-DBD Plasma Treatment

To understand the effect of c-DBD plasma-treated water on plant defense and antioxidant potential related molecular gene expression mechanisms in onion during the early seedling stage particularly in relation to dormancy release and improved growth we conducted qRT-PCR analysis.

**Figure 5:**
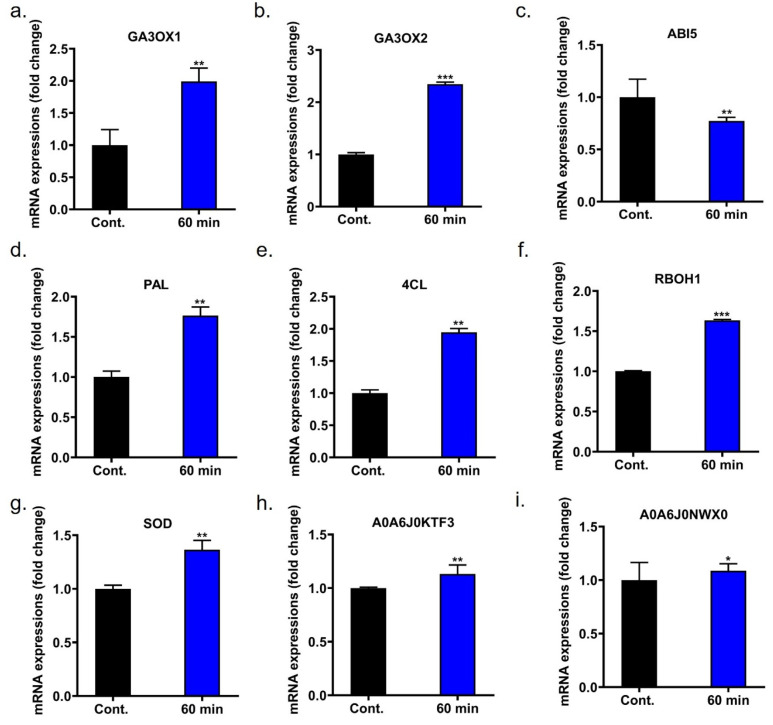
Molecular analysis of onion seedlings subjected to control and 60 min of c-DBD plasma treatments. Panels (**a**–**c**) depict gene expression patterns linked to both growth-promoting and growth-inhibiting functions in the onion seedlings under 60 min of c-DBD plasma treatment conditions, including the control and 60 min, (**a**) GA3OX1, (**b**) GA3OX2, (**c**) ABI5. Panels (**d**,**e**) display expression of genes in the phenylpropanoid pathway, (**d**) PAL, (**e**) 4CL. Panels (**f**,**g**) present genes involved in reactive oxygen species (ROS) signaling, (**f**) RBOH1, (**g**) SOD. Panels (**h**,**i**) show protein synthesis-related genes, (**h**) A0A6J0KTF3 and (**i**) A0A6J0NWX0 Statistical differences between treatment groups are indicated as * *p* < 0.05, ** *p* < 0.01, and *** *p* < 0.001 and “ns” denoting no significant difference compared to the control group.

Gibberellic acid (GA), a key growth-promoting hormone, plays a pivotal role in breaking dormancy, promoting shoot elongation, and driving cell division. In this study, GA3-oxidase genes (especially *GA3OX1* and *GA3OX2*) were upregulated by 1.99- and 2.34-fold, respectively, after 60 min of plasma treatment (Figure 5a,b). These genes are vital for the biosynthesis of bioactive gibberellin. In contrast, the expression of *ABA Insensitive 5 (ABI5)*, a crucial component of the abscisic acid (ABA) signaling cascade, was significantly reduced 0.77-fold in the 60 min plasma-treated group, indicating decreased sensitivity to ABA-induced inhibition of growth (Figure 5c). As shown in Figure 5d,e, the phenylpropanoid pathway enzymes, *PAL* and *4CL*, which are key to the biosynthesis of a wide range of secondary metabolites, including lignin, flavonoids, and anthocyanins, showed altered expression after plasma treatment. Both enzymes were upregulated by 1.77- and 1.97-fold throughout the seedling stage. In addition, *Respiratory Burst Oxidase Homolog 1 (RBOH1)* and *Superoxide Dismutase (SOD)* play important regulatory roles in reactive oxygen species (ROS) signaling. Compared to the untreated group, RBOH1 and SOD genes were upregulated by 1.36- and 1.63-fold after 60 min of c-DBD plasma treatment, as shown in Figure 5f,g. Plasma treatment further stimulated gene expression in protein synthesis pathways, particularly increasing the expression of *arginine-tRNA ligase (A0A6J0KTF3)* and *lectin-B-like (A0A6J0NWX0)* (Figure 5h,i).

### 2.13. Properties of PTW

**Figure 6:**
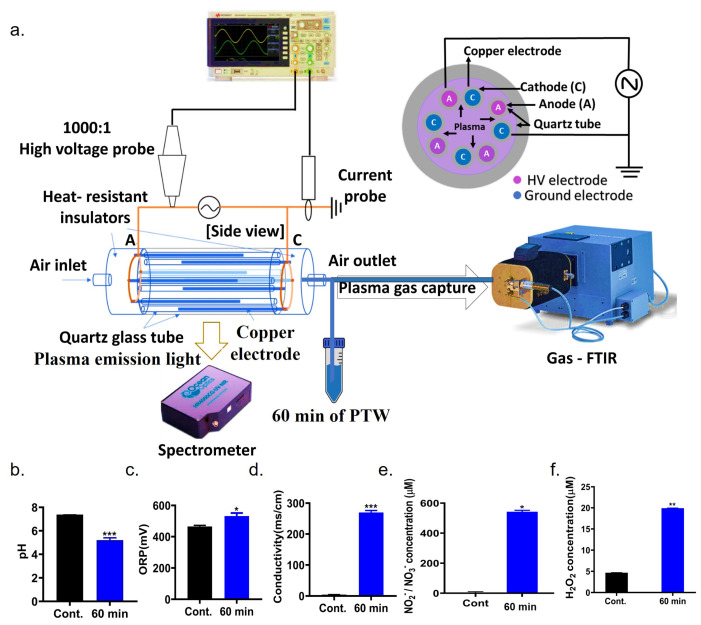
Schematic diagram of c-DBD plasma and its physicochemical properties. (**a**) The experimental setup of multi-electrode c-DBD plasma for generation of plasma-treated water (PTW). (**b**–**f**) Evaluation of fundamental physicochemical characteristics of the prepared PTW, including pH, oxidation reduction potential (ORP), electrical conductivity, and NO_2_^−^/NO_3_^−^ content. Statistical differences between treatment groups are indicated as * *p* < 0.05, ** *p* < 0.01, and *** *p* < 0.001. Deionized water was treated by c-DBD plasma around 60 min.

When c-DBD-induced plasma interacts with deionized water, it generates a range of reactive oxygen and nitrogen species (RONS) within the liquid. A previous study has shown that nitrate ions (NO_3_^−^) account for only about 5% of the total RONS, with nitrite ions (NO_2_^−^) comprising the majority. In contrast, hydrogen peroxide (H_2_O_2_) appears in extremely low concentrations or is not detectable at all. Owing to the dominant presence of nitric oxide (NO), this plasma-treated water is commonly referred to as nitric oxide-enriched water. To evaluate PTW suitability for agricultural use, different physicochemical properties including pH, oxidation-reduction potential (ORP), conductivity, and concentrations of nitrogen-based ions (NO_2_^−^ and NO_3_^−^) were measured immediately after 60 min c-DBD plasma exposure. The pH values for both control and 60 min PTW were between 7.38 and 5.20 (Figure 6b), a range that supports optimal seed germination and plant growth. Notably, 60 min of plasma treatment altered the ORP and conductivity of the water. ORP increased from 465.30 mV in the control to 531.97 mV in PTW, while conductivity rose from 4.29 to 269.61 mS/cm, as shown in Figure 6c,d. These changes are attributed to the presence of reactive nitrogen species. The total concentration of NO_2_^−^ and NO_3_^−^ (collectively referred to as NOx) in PTW was approximately 542.09 µM (Figure 6e). The concentration of H_2_O_2_ increased from 4.67 µM in the control to 19.92 µM following 60 min of plasma treatment.

## 3. Discussion

c-DBD plasma technology has emerged as a promising method in agriculture, particularly for enhancing seed germination and seedling development. It can generate reactive species such as O, •OH, NO, NO_2_, NO_3_, O_2_•^−^, and H_2_O_2_, [11,17], which play crucial roles in regulating cellular metabolism [10]. In this study we mainly focus on NO-dominated reactive species like NO, NO_2_^−^, and NO_3_^−^ in the gas and liquid phases. Water absorption studies have indicated that PTW-treated seeds exhibit higher imbibition rates than untreated seeds, suggesting enhanced water flow into the seeds (Figure 2a) [10]. PTW treatment can alter the structural integrity of the onion seed testa, leading to increased water uptake and potentially enhancing seed germination (Figure 2b,c) and early seedling development. However, the optimal exposure time and conditions are crucial to avoid excessive damage that could impede seed viability. Water and reactive nitrogen species synergistically stimulate enzymatic activities for seed germination. NO and its derivatives, such as nitrogen dioxide (NO_2_) and nitrate (NO_3_^−^), can interact with the seed coat, leading to chemical modifications that affect its permeability [10]. These interactions can result in the formation of micro-pores or the alteration of surface chemistry, facilitating water uptake and gas exchange, which are critical for germination. Furthermore, NO can act as a signaling molecule, modulating the expression of genes involved in seed dormancy and germination processes [12,14]. In total, 60 min of c-DBD plasma exposure exhibits higher germination and seedling growth.

Phenotypical analysis revealed that seedlings from 60 min of plasma-treated seeds have increased shoot, root lengths, number, fresh and dry weights compared to controls, as shown in Figure 3a–h. These enhancements are attributed to the activation of phytohormone catabolism and improved nutrient uptake by c-DBD plasma [10]. Biochemical profiling has also shown elevated levels of NO_2_^−^, NO_3_^−^, and H_2_O_2_ in 60 min of c-DBD plasma-treated seedlings, as shown in Figure 4d–f. H_2_O_2_ and NO function as signaling molecules that promote cell growth and activate defense mechanisms [13,19]. The upregulation of reactive species stimulates the antioxidant machinery in seedlings to maintain cellular integrity [13,20]. In addition, 60 min of c-DBD plasma treatment also elevated the levels of chlorophyll, sugars and proteins in onion seedlings as shown in Figure 4a–c [21,22,23]. Notably, increased catalase (CAT) activity has been observed, indicating an enhanced breakdown of hydrogen peroxide (H_2_O_2_) into water and oxygen. This reduction in H_2_O_2_ levels is crucial, as excessive H_2_O_2_ can lead to oxidative damage [24]. By modulating H_2_O_2_ concentrations, catalase indirectly supports processes such as root development, cell wall expansion, and growth hormone signaling, ensuring optimal conditions for seedling growth and development (Figure 4f,j) [25]. Additionally, lower ascorbate levels suggested enhanced activity of ascorbate peroxidase and ascorbate oxidase enzymes [26]. Reduced malondialdehyde (MDA) content [27] and increased proline levels further confirm the stimulate antioxidant system in 60 min of c-DBD-plasma-treated seedlings, as shown in Figure 4g–i [24]. These biochemical alterations underscore the efficacy of c-DBD plasma treatment in enhancing plant physiological processes, leading to improved growth and resilience under severe environmental stress.

Under 60 min of c-DBD plasma treatment, onion seedlings exhibited significant changes in gene expression associated with growth regulation, stress response, and metabolic pathways. Real-time quantitative qRT-PCR analysis revealed upregulation of growth-promoting genes such as *GA3OX1* and *GA3OX2* (Figure 5a,b), which are involved in gibberellin biosynthesis and play critical roles in stem elongation and seedling development. Conversely, the expression of *ABI5* (Figure 5c), a gene linked to abscisic acid signaling and growth inhibition, was also elevated, indicating a complex regulatory balance between growth promotion and inhibition [28,29,30]. In the phenylpropanoid pathway, key genes like *PAL* and *4CL* show increased expression (Figure 5d,e), suggesting enhanced synthesis of secondary metabolites that contribute to structural integrity and defense mechanisms [31,32,33]. Additionally, genes involved in reactive oxygen species (ROS) signaling, such as *RBOH1* and *SOD* (Figure 5f,g), are upregulated, reflecting an activated antioxidant defense system [34,35] to mitigate oxidative stress induced by plasma treatment.

Furthermore, genes related to protein synthesis, including *A0A6J0KTF3* and *A0A6J0NWX0* (Figure 5h,i), exhibit increased expression levels, indicating an overall boost in metabolic activity and protein production necessary for growth and adaptation [36]. These molecular alterations underscore the multifaceted effects of c-DBD plasma treatment, enhancing growth-related pathways while simultaneously activating stress-responsive mechanisms, thereby promoting resilience and development in onion seedlings.

Moreover, c-DBD plasma treatment modifies the seed coat, enhancing water and gas exchange, which in turn stimulates the generation of reactive species within the seeds. These reactive species act as signaling molecules by stimulating enzymatic activities like amylase and initiating germination signaling cascades [37]. Moreover, plasma treatment influences gene expression related to growth, stress response, and metabolic pathways, leading to improved seedling growth and resilience [24]. These findings highlight the potential of c-DBD plasma technology as an effective tool for enhancing seed germination and seedling development in agriculture.

## 4. Materials and Methods

### 4.1. Properties of c-DBD Plasma System

A multi-electrode cylindrical dielectric barrier discharge (c-DBD) was used in this study to generate large quantities of reactive nitrogen species (RNS), as shown in Figure 6a. Four pairs of line-shaped electrodes are arranged opposite each other within the outer quartz tube. The quartz tube has a diameter of about 30 mm and a total length are 300 mm. The electrode is enclosed in a small quartz tube with a 3 mm diameter and covered by dielectric material. The electrodes, each 10 mm long, and positioned directly opposite their counterparts, facilitating plasma generation between them. A (anode) and C (cathode) electrodes were connected with high voltage inverter of V_pp_ = 20 kV. Plasma’s electrical properties were measured using an oscilloscope (WaveSurfer 434, Teledyne LeCroy, Chestnut Ridge, NY, USA), along with a high-voltage probe (Tekronix, P6015A, Beaverton, OR, USA) and a current probe (LeCroy, CP030, Chestnut Ridge, NY, USA). The concentration of the reactive nitrogen species in the gas phase was measured using gas-FTIR spectrometer (Fourier transform infrared, MATRIX-MG5 in Bruker Co., Billerica, MA, USA). Plasma temperatures and densities have been calculated using a nitrogen collisional radiation (CR) model using a spectrometer (HR4000CG-VIS-IR, Ocean optics Co., Orlando, FL, USA) of optical emission spectroscopy (OES). The c-DBD system operated using ambient air at a controlled flow rate of 1.0 L per minute (LPM) to aid the gas movement. To generate plasma-treated water (PTW), 50 mL of deionized water was continuously exposed to the gas during 60 min plasma generation.

### 4.2. Physicochemical Characteristics of PTW

The physicochemical properties of plasma-treated water (PTW) were assessed immediately after exposure to air plasma. Total nitrite (NO_2_^−^/NO_3_^−^) content was quantified using a Griess assay-based commercial kit (D2NO-100; Bioassay Systems, Waltham, MA, USA). Hydrogen peroxide (H_2_O_2_) concentration was measured using the QuantiChrom™ Peroxide Assay Kit, following the manufacturer’s instructions. Absorbance was recorded at 585 nm using a microplate reader (Synergy HTX Multi-Mode Reader, BioTek Instruments, Winooski, VT, USA), and H_2_O_2_ levels were quantified based on a standard calibration curve. The pH, conductivity, and oxidation reduction potential (ORP) were measured using a pH spear probe (Eutech Instruments, Paisley, UK), a CON30 Tester, and an ORP30 Tester (Clean Instruments, Shanghai, China), respectively [31].

### 4.3. Assessment of Water Uptake

The water uptake of onion seeds was utilized by weighing about 10 g of seeds both before and after soaking in either deionized water (control) and plasma-treated water (PTW). At selected time intervals (0, 2, 4, 6, 8, and 10 h), the seeds were removed, gently blotted with absorbent paper, and weighed. The water absorption rate (*Wa*) for each group was calculated using the formula below:

$$Wa\left(\%\right)=\frac{W1-W0}{W0}\;\times 100\%\;$$

where *W*0 represents the initial weight of the seeds, and *W*1 denotes the weight of the seeds after water absorption at each specified time interval [18].

### 4.4. Assessment of Seed Germination Efficiency and Plant Growth

Randomly selected *Allium cepa* (*onion*) seeds were divided into 5 groups, each containing 100 seeds. Initially these groups were subjected to different periods of plasma treatments: 15 min, 30 min, 60 min, and 120 min. An untreated seed served as control (Cont.). Among all treatment conditions, two groups were chosen for the entire set of experiments. To assess seed germination, both treated and untreated seeds were placed on sterile paper towels within plant culture dishes. Each dish contained 20 seeds, and experiments were performed in triplicate, totaling 60 seeds per treatment. The dishes were moistened with 3 mL of deionized water and placed in a growth chamber maintained at 25 ± 2 °C temperature, 76 ± 5% relative humidity, and a 16 h light/8 h dark cycle, with light provided at 44 W m^−2^ by a Philips fluorescent tube. Water was supplied twice a week to ensure consistent moisture levels during the germination process. On the third day, germination was determined by counting the number of sprouted seeds, and the germination percentage was calculated according to the previous study [24]. Once germinated, the seedlings were transferred into pots filled with an equal mixture of soil and vermiculite (1:1). To prevent desiccation, deionized water was added twice a week. Fifteen days after the plasma treatment, both control and treated plants were harvested. At the time of harvest, shoot lengths were recorded, and leaf samples were collected, flash-frozen in liquid nitrogen, and stored at −80 °C until further examination.

### 4.5. Determination of Phenotypical Traits

Plant growth-related variables for onion were determined 15 days after planting by measuring shoot and root lengths as well as fresh and dry weights. Lengths were measured using a meter scale.

For the determination of dry matter content, fresh onion seedlings were first rinsed with deionized water to remove surface debris, then gently blotted with tissue paper to eliminate excess moisture. The fresh weight (FW) of each whole seedling was recorded using a digital weighing balance (Mettler Toledo, Columbus, OH, USA). The samples were then placed in a hot air oven at 65 ± 2 °C and dried to a constant weight for 48 h. After drying, the samples were cooled in a desiccator and weighed to determine the dry mass.

Based on the collected onion bulb images, the bulb area was measured using ImageJ(1.54k) software. The hardness of the bulb was measured using a penetrometer (High Quality LX-A/C/D Hardness Tester, ETOPOO, Shenzhen, China).

### 4.6. Determination of Chlorophyll Content

A total of 100 mg of leaf sample was collected from 5 different onion plants, and the sample were ground with 5 mL of 80% acetone solution to determine chlorophyll content. The solution mixtures were placed into test tubes and gently inverted several times to facilitate pigment extraction. Samples from both the control and 60 min plasma-treated groups were covered with aluminum foil and left at room temperature until the leaf fragments became fully transparent. The filtered extracts were then used to measure the absorbance of 100 μL aliquots at wavelengths of 470 nm, 646 nm, and 663 nm, using a Synergy HTX Multi-Mode Reader (BioTek Instruments, VT, USA). These absorbance values were then applied to calculate the contents of chlorophyll a, chlorophyll b, total chlorophyll (a + b), using formulas according to previous research [38]. Each analysis was performed in at least 4 biological replicates (n = 4).

### 4.7. Determination of Total Soluble Protein

Onion shoots and roots were first thoroughly rinsed with deionized water and then finely chopped. A 100 mg of each tissue were frozen with liquid nitrogen and homogenized in 1 mL of phosphate-buffered saline (PBS). The homogenates were centrifuged at 13,000× *g* for 20 min. Following centrifugation, the supernatant was carefully transferred into clean test tubes. The total soluble protein content was assessed using the DC protein assay kit (Bio-Rad, Hercules, CA, USA), with bovine serum albumin serving as a standard [39].

### 4.8. Determination of Total Soluble Sugar

A total of 300 mg of onion seedlings (including root, shoot, and bud) were ground using a mortar and pestle. The resulting extract was then collected using a dropper to measure the soluble sugar content witth a portable Khalder refractometer (FHI Korea Co., Seoul, Republic of Korea). The refractive index obtained values were converted to °Brix. Measurements were carried out at room temperature, and the prism surface of the refractometer was carefully cleaned with deionized water after each test to ensure reliable results.

### 4.9. Determination of Reactive Species

To measure the levels of nitrite and nitrate ion, 150 mg of onion seedling were ground in 1.5 mL of cold glacial acetic acid using a mortar and pestle. The homogenized samples were centrifuged at 12,000× *g* for 15 min at 4 °C to remove any residual cell debris. The supernatants were carefully collected and transferred into fresh centrifuge tubes. Total NO_2_^−^ and NO_3_^−^ levels were determined using a Griess assay-based commercial kit (D2NO-100; Bioassay Systems, USA). The absorbance was measured at 540 nm from a microplate reader (Synergy HTX Multi-Mode Reader from Bio-Tek Instruments, Winooski, VT, USA), using a standard curve for quantification. To prepare thehomogenate, 300 mg of onion seedling were ground with liquid nitrogen and mixed with 5 mL (0.1% *v*/*v*) of trichloroacetic acid solution. The solution was then centrifuged at 1500× *g* for 10 min at 4 °C. The supernatant was collected and mixed with 500 µL of 10 mM potassium phosphate buffer (pH 7.0) and 1 mL of 1 M potassium iodide. The amount of hydrogen peroxide (H_2_O_2_) was determined according to QuantiChrom^TM^ Peroxide assay kit. The absorbance was measured at 585 nm from a microplate reader (Synergy HTX Multi-Mode Reader from Bio-Tek Instruments, Winooski, VT, USA), using a standard curve for quantification [40].

### 4.10. Malondialdehyde Content

To determine lipid peroxidation in cellular membranes, the thiobarbituric acid reactive substances (TBARS) assay was used, which quantified malondialdehyde (MDA). A total of 100 mg of onion seedlings was homogenized in 3 mL of 0.1% trichloroacetic acid (TCA) using a mortar and pestle. After that, the homogenate was centrifuged at 13,000× *g* for 10 min at 4 °C. After centrifugation, 1.0 mL of supernatant was mixed with 4.0 mL of TBARS solution and incubated at 100 °C for 30 min. Upon completion of the reaction, the solution mixture was cooled to room temperature. Fluorescence was measured using a microplate reader at 540 nm excitation and 600 nm emission wavelengths in a black 96-well plate. All analyses were carried out in a minimum of 3 replicates to ensure consistency and accuracy.

### 4.11. Proline Content

The quantification of free proline in onion tissues was performed according to a previous study. A total of 0.5 g onion seedlings was finely powdered in liquid nitrogen and then mixed with 3% sulfosalicylic acid to extract proline. The resulting homogenate was centrifuged at 10,000× *g* for 5 min to remove particulate matter. To 500 μL of the collected supernatant, equal volumes of glacial acetic acid and ninhydrin reagent (500 μL each) were added to a clean test tube. The mixture was incubated in a 90 °C water bath for 50 min. After incubation, the tubes were immediately transferred to an ice bath for rapid cooling. An equal amount of toluene was added, and the mixture was vortexed thoroughly. The absorbance of the toluene phase (upper layer), which contained the proline, was then measured at 520 nm using a microplate reader (Biotech, VT, USA).

### 4.12. Ascorbate Content

A colorimetric Vitamin C Test Kit (Elabscience, Houston, TX, USA) was utilized to measure the ascorbic acid in onion seedlings. The test follows a two-step procedure that starts when ascorbic acid converts Fe^3+^ to Fe^2+^, followed by Fe^2+^ generating a colored compound with phenanthroline. The absorbance of the final solution was recorded at 536 nm using a microplate reader.

### 4.13. Catalase Activity

The Enzyme Chrom™ Catalase Assay Kit (ECAT-100, BioAssay Systems) was employed to measure catalase (CAT) activity in onion seedlings, following the manufacturer’s instructions. One unit of enzyme activity was defined as the quantity of CAT needed to decompose 1 μmol of hydrogen peroxide (H_2_O_2_) per minute at pH 7 and room temperature. Absorbance was determined by a microplate reader at 570 nm to measure CAT activity, which was expressed in units per liter (U/L) [24].

### 4.14. qPCR Analysis

Total RNA was isolated from 200 mg of onion seedlings using TRI Reagent (Sigma-Aldrich, Seoul, Republic of Korea), following the manufacturer’s instructions. We measured the extracted RNA quality through a microplate reader that examined the absorbance ratio ranging from 260 to 280 nm. Quantitative real-time PCR (qRT-PCR) was performed using the CFX Connect Real-Time PCR Detection System (Bio-Rad, Seoul, Republic of Korea) with SYBR Green Master Mix (Bio-Rad). The primer sequences used in this study are described in Table 1.

### 4.15. Statistical Analysis

The data obtained from three independent experiments (n = 3) were analyzed and displayed as mean ± standard error (SE) using Microsoft Excel (Office 365). Statistical significance was determined using Student’s *t*-test, with *p*-values below 0.05 considered statistically significant. Significance levels were indicated by asterisks: * for *p* < 0.05, ** for *p* < 0.01, and *** for *p* < 0.001.

## 5. Conclusions

Cylindrical dielectric barrier discharge (c-DBD) plasma-treated water represents a promising eco-friendly technology with broad potential in sustainable agriculture. This study is the first to elucidate the effects of c-DBD plasma on onion (*Allium cepa*) seed germination and early seedling growth at molecular and physiological levels. Plasma treatment effectively delivers reactive nitrogen species such as nitric oxide (NO) to plants, accelerating dormancy release and modulating hormonal balance by increasing gibberellins and suppressing abscisic acid. Simultaneously, it activates phenolic synthesis and antioxidant systems, enhancing oxidative stress tolerance. These findings provide critical insight into how c-DBD plasma not only serves as a physical treatment but also orchestrates reactive oxygen and nitrogen species (ROS/RNS) signaling pathways to reprogram metabolic networks and hormone homeostasis, thereby promoting development and stress resilience during early plant growth stages. Future research should focus on deciphering the detailed molecular mechanisms underlying plasma-induced changes in gene expression and epigenetic regulation related to growth and stress responses. Crop-specific optimization of plasma treatment protocols and engineering advances for scalable, field-deployable plasma devices are also essential. Furthermore, long-term field trials are necessary to assess the sustained impact of plasma treatment on crop health, yield, and resilience under variable environmental conditions. By addressing these areas, the full potential of c-DBD plasma technology can be unlocked, advancing sustainable and smart agricultural practices. This technology holds great promise as an innovative alternative to conventional agrochemicals, contributing to eco-friendly crop production and global food security.

## Figures and Tables

**Table 1:** List of primers used for gene expression.

Gene	Forward	Reverse
*Glyceraldehyde* *3-phosphate dehydrogenase* *(GAPDH) endogenous gene*	AACATTATCCCCAGCAGCAC	TAGGAACTCGGAATGCCATC
*Gibberellin 3-beta-dioxygenase2* *(GA_3_OX1)*	GTCGGAGTCCTTCCAACTGAA	GGCCGTTGGATAATATGTGG
*Gibberellin 3-beta-dioxygenase2* *(GA_3_OX2)*	TTTGCCAAGCAAATGTGGTA	TCTGGACCAGCCCATTCTAC
*Abscisic Acid-Insensitive 5 (ABI5)*	ACCACCGCATGATCAAGAAC	GGTGGTTTAGTTCGGCTTCA
*Phenylalanine lyase (PAL)*	CCTCAACATCACTCCATGCC	CTCAAAGAAGGACGGGACGC
*4-Coumarate:CoA ligase (4CL)*	TCGTAGACAGGGTGAAGGAGC	CTTCACCAGCAGCCTCATCT
*Respiratory Burst Oxidase Homolog 1 (RBOH1)*	CGAAGCATTTCTCGCAAG	TTGAGTCCACGAAGAGCCTT
*Superoxide dismutase [Cu-Zn] 1 (SOD)*	GACCACATTACAATCCTGCTG	CAATGATGGACTGTGGACCAG
*Arginine-tRNAligase (A0A6J0KTF3)*	ATGCAATGGGTCTCTGGTCA	GCCCATGTGTCAATTCCGTT
*Lectin-B-like (A0A6J0NWX0)*	ATGTTCCCGTCCTGCAGT	TGGTGGGATTAGGAGGAGTG

## Data Availability

Data are available from the corresponding author upon reasonable request.

## Supplementary Materials

The following supporting information can be downloaded at: https://www.mdpi.com/article/10.3390/ijms26157256/s1.
