# Supplementary material for: Investigating Seed Germination, Seedling Growth, and Enzymatic Activity in Onion (Allium cepa) Under the Influence of Plasma-Treated Water
Source: Int J Mol Sci. 2025 Jul 27;26(15):7256. doi: 10.3390/ijms26157256 (PMC12347931; doi:10.3390/ijms26157256)
Supplement: Supplementary file 1 [file ijms-26-07256-s001.zip › ijms-3751928-supplementary.pdf]

International Journal of
Molecular Sciences

MDPI

Supporting information

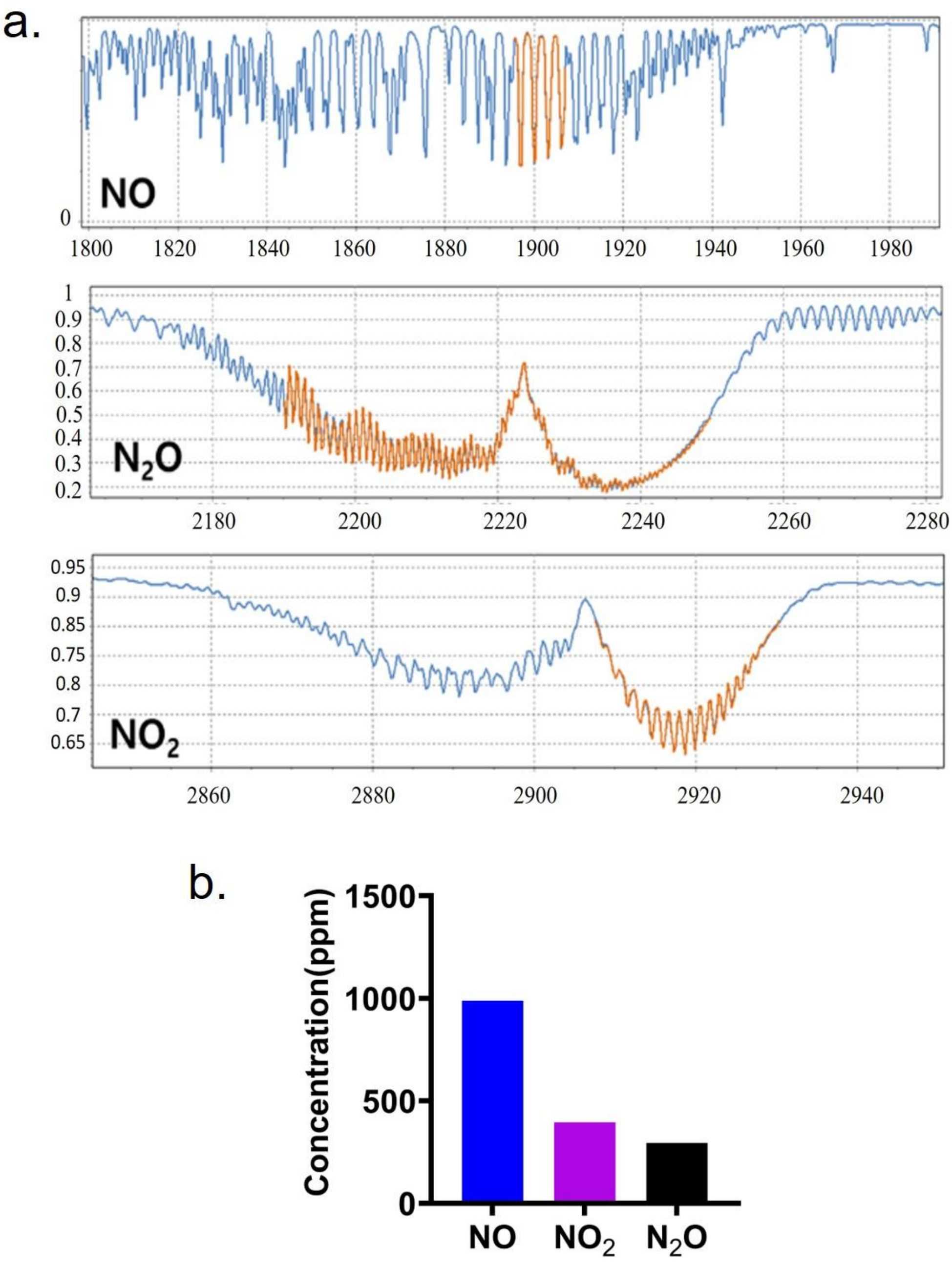

Figure S1. (**a**) The time-dependent gas concentration of multi-electrode c-DBD plasma, (**b**) specific absorption spectra for NO, N_2O, and NO_2 extracted from the total spectra.
